# Development of an *ex vivo* respiratory pediatric model of bronchopulmonary dysplasia for aerosol deposition studies

**DOI:** 10.1038/s41598-019-42103-2

**Published:** 2019-04-05

**Authors:** Yoann Montigaud, Sophie Périnel, Jean-Christophe Dubus, Lara Leclerc, Marie Suau, Clémence Goy, Anthony Clotagatide, Nathalie Prévôt, Jérémie Pourchez

**Affiliations:** 1 Mines Saint-Etienne, Univ Lyon, Univ Jean Monnet, INSERM, U 1059 Sainbiose, Centre CIS, F – 42023 Saint-Etienne, France; 2 INSERM U 1059 Sainbiose, Université Jean Monnet, F-42023 Saint-Etienne, France; 3 CHU Saint-Etienne, Saint-Etienne, F-42055 France; 4 Médecine infantile, pneumo-allergologie, CRCM & CNRS, URMITE 6236, Assistance publique-Hôpitaux de Marseille, 13385 Marseille cedex 5, France

## Abstract

Ethical restrictions are limitations of *in vivo* inhalation studies, on humans and animal models. Thus, *in vitro* or *ex vivo* anatomical models offer an interesting alternative if limitations are clearly identified and if extrapolation to human is made with caution. This work aimed to develop an *ex vivo* infant-like respiratory model of bronchopulmonary dysplasia easy to use, reliable and relevant compared to *in vivo* infant data. This model is composed of a 3D-printed head connected to a sealed enclosure containing a leporine thorax. Physiological data and pleural-mimicking depressions were measured for chosen respiratory rates. Homogeneity of ventilation was assessed by ^81m^krypton scintigraphies. Regional radioaerosol deposition was quantified with ^99m^technetium-diethylene triamine pentaacetic acid after jet nebulization. Tidal volumes values are ranged from 33.16 ± 7.37 to 37.44 ± 7.43 mL and compliance values from 1.78 ± 0.65 to 1.85 ± 0.99 mL/cmH_2_O. Ventilation scintigraphies showed a homogenous ventilation with asymmetric repartition: 56.94% ± 9.4% in right lung and 42.83% ± 9.36 in left lung. Regional aerosol deposition in lungs exerted 2.60% ± 2.24% of initial load of radioactivity. To conclude the anatomical model satisfactorily mimic a 3-months old BPD-suffering bronchopulmonary dysplasia and can be an interesting tool for aerosol regional deposition studies.

## Introduction

Bronchopulmonary dysplasia (BPD) is one of the most common pathology of pre-term newborns^1–4^. This pathology is characterized by the need of supplemental oxygenation or ventilation for at least 28 days of age. Severity is assessed with the remaining need of oxygen at respectively 36 and 56 weeks post-menstrual age (PMA) for infants born before or after 32 weeks of gestation. Physiopathologically, BPD represents the interruption of lung development before the saccular stage, starting at 32 weeks PMA, and corresponds to the formation and architectural development of alveoli^5^. Risk factors to develop BPD are numerous: internal factors (prematurity, gender, genetics, *in utero* tobacco exposure), iatrogenic factors (hyperoxia related to mechanical ventilation or/and oxygen supplementation, blood transfusion) or external factors (antenatal or/and postnatal infection)^3,6–8^.

Treatments of BPD include prophylactic and symptomatic approaches such as aerosol delivery of surfactant, ante- and/or postnatal administration of corticosteroids and ventilation strategies^6,9–11^. Despite existing therapeutic options, there are still long-term outcomes of BPD with high rates of cognitive or behavioral impairments and reduced lung function^12^, even impacts on motor skills, cognitive and language developments^6,13^. Administration of corticosteroids, by systemic or inhaled administration route, remain one of the main strategy to limit outcomes of BPD and to wean infants from ventilation^14^. Moreover, current inhaled treatments were tested against systemic corticoids. No statistical differences could be found between the two administration routes^10^. Inhaled corticotherapy could be use as a prophylactic treatment on very low birth weight preterms but failed to show significant efficacy^15,16^ and as a symptomatic treatment^17^. However, these approaches are known to impair neurologic development of newborn^18^ and infants and to increase relative risk associated to death for extremely preterm newborns^19^. This could be due to a non-optimized nebulization technology, leading to an uncontrolled or uneven deposition of drugs in the lungs. Hence, there is an unmet medical need in the cartography and quantification of pulmonary deposition of aerosolized drugs. All things considered, considering the well known side effects of systemic administration, local delivery of drugs to the lungs are expected to be an interesting route of administration as an alternative to systemic therapies in the management of BPD.

The aerosol deposition cartography depend on airborne particles properties (*e*.*g*. aerodynamic size, hydroscopic properties, electrical surface charge, etc.) and also on anatomic and physiologic (*e*.*g*. diameter of airways, breathing frequency, tidal volume, etc.)^20,21^. Nonetheless, as radiolabeled aerosols are the gold standard to assess regional aerosol deposition of inhaled particles within lungs^22^ *in vivo* human studies are still scarce due to ethical restrictions. Therefore, as an alternative, *in vivo* deposition studies using rodent models are frequent^23,24^. For example, *in vivo* studies on rats are common and different models were developed especially for BPD studies^25^. Other *in vivo* animal models exist, such as baboon^26^, macaque^27^, piglet^28^ or rabbit^29^, and could be used as infant-like model for aerosol regional deposition studies. However, using animal models is expensive and time-consuming (*i*.*e*. very high cost involved in breeding and housing, length of protocols for animal experiments). Also, there are remaining discrepancies to mimic infants/newborns respiratory tracts, such as breathing frequencies or anatomy of lungs. Finally, *in vivo* human studies of regional deposition assessed by radiolabeled aerosols are focused mainly on adults, thus infant/newborn features are addressed by mathematical modeling^30^. Deposition models for inhaled particles are based on extrapolation of data from young adults to avoid bias of growth and aging^31^. These mathematical models predictions trends to overestimate tracheobronchial deposition an to underestimate pulmonary one for children compared to adult^32^. Hence, a greater pulmonary dose could be administered to infants due to architecture of airways, lung surface area or breathing pattern^33^. This lack of data concerning inhalation studies focused on infants requires relevant and reliable respiratory models to perform inhalation studies.

Therefore, this study aimed to develop an *ex vivo* respiratory model of infants, which could be a useful tool for the study of aerosol treatments of BPD. Based on previous work of our laboratory^34,35^, this chimeric model is composed of a 3D-printed upper airways replica of the Sophia Anatomical Infant Nose-Throat (SAINT) model connected to an *ex vivo* leporine respiratory thorax placed in a sealed instrumented enclosure specifically designed for that purpose. Leporine lungs are ventilated by applying negative pressure in the enclosure to mimic pleural depression and passive ventilation. Objectives of this work were to validate the likelihood of this innovative *ex vivo* respiratory model in comparison with infants’ respiratory physiology and existing alternative animal models used in literature. This study is divided into 3 main parts: i) breathing pattern and respiratory parameters, ii) assessment of ventilation and iii) regional aerosol deposition within respiratory tract.

## Results and Discussion

### Breathing pattern and respiratory parameters

**Figure 1:**
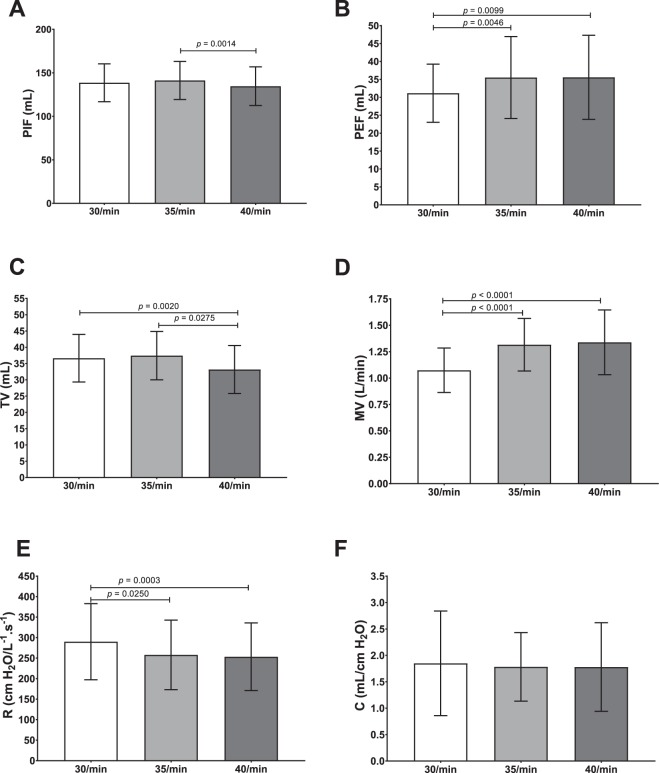
Comparison of respiratory parameters (mean ± SD; n = 38), p < 0.05 is considered as significant. (**A**) Peak inspiratory flow (PIF). (**B**) Peak expiratory flow (PEF). (**C**) Tidal volume (TV). (**D**) Minute ventilation (MV). (**E**) Resistances (R). (**F**) Compliance (C).

Validation of respiratory parameters was performed using 38 respiratory models with various breathing frequencies in the range 30–40 cycles/min. For each respiratory rate, 6 respiratory parameters were assessed: peak inspiratory flow (PIF), peak expiratory flow (PEF), tidal volume (TV), minute-ventilation (MV), resistances (R) and compliance (C). These parameters were calculated for each respiratory model (*n* = 38) by collecting data from at least 50 breathing cycles to calculate mean values for each respiratory model. Table 1 and Fig. 1 report mean values and the dispersion of the experimental data collected for each of the 38 respiratory models at various breathing frequencies (30, 35 and 40 cycles/min).

**Table 1:** Comparison of respiratory parameters and breathing pattern for each respiratory rate (n = 38; data are presented as mean ± SD [confidence interval 95%]).

RR	30 cycles/min	35 cycles/min	40 cycles/min
RR (cycles/min)	29.35 ± 0.83 [29.08; 29.62]	35.23 ± 1.16 [34.85; 35.61]	40.31 ± 0.84 [40.03; 40.58]
IT (s)	0.46 ± 0.09 [0.43; 0.49]	0.46 ± 0.08 [0.43; 0.49]	0.43 ± 0.07 [0.41; 0.46]
ET (s)	1.58 ± 0.10 [1.54; 1.61]	1.24 ± 0.09 [1.21; 1.27]	1.06 ± 0.08 [1.03; 1.08]
PIF (mL)	138.6 ± 21.75 [131.4; 145.7]	141.2 ± 21.93 [134.0; 148.4]	134.6 ± 22.23 [127.3; 142.0]
PEF (mL)	31.14 ± 8.13 [28.47; 33.81]	35.52 ± 11.42 [31.77; 39.28]	35.59 ± 11.74 [31.73; 39.45]
TV (mL)	36.63 ± 7.31 [34.23; 39.03]	37.44 ± 7.43 [34.99; 39.88]	33.16 ± 7.37 [30.74; 35.58]
MV (L/min)	1.07 ± 0.21 [1.01; 1.14]	1.32 ± 0.25 [1.23; 1.40]	1.34 ± 0.31 [1.24; 1.44]
R (cmH_2_O/L^−1^.s^−1^)	290.1 ± 92.77 [320.6; 259.6]	258.0 ± 84.70 [285.8; 230.1]	253.5 ± 82.50 [280.6; 226.4]
C (mL/cmH_2_O)	1.85 ± 0.99 [1.52; 2.17]	1.78 ± 0.65 [1.57; 2.00]	1.78 ± 0.84 [1.50; 2.06]

Respiratory rate (RR), inspiratory time (IT), expiratory time (ET), peak inspiratory flow (PIF), peak expiratory flow (PEF), tidal volume (TV), minute ventilation (MV), resistances (R), compliance (C).

### Breathing frequency and inspiratory/expiratory time

IT and ET are easily tunable. IT was set at 0.4 s and then ET values vary (set at 1.6 s, 1.3 s and 1.1 s respectively) in order to obtain chosen breathing frequencies (*i*.*e*. 30, 35 and 40 cycles/min respectively). This range of respiratory rates (RR) is similar to those found in infants suffering from BPD, 43.7 ± 13.5 cycles/min^36^. Experimental data collected demonstrated that, as awaited, no significant difference (*p* = 0.0568) were obtained for the experimental IT when compared to aimed value. RR and ET are close from aimed values meaning that this model is tunable at will for these breathing frequencies.

### Peak inspiratory/expiratory flow

PIF values varied between 134.6 ± 22.23 mL and 141.2 ± 21.93 mL, while PEF values spread from 31.14 ± 8.13 mL to 35.59 ± 11.74 mL (Table 1). PIF values are around two times higher than values reported in literature. However, PEF values are almost 2 times lower than *in vivo* data^37^. Latzin *et al*.^37^ study focused on BPD-suffering infants (113–121 days of age), they found PIF values of 76 ± 16 mL/s and PEF values of 70 ± 17 mL/s. These differences could be explained by the tuning of IT and ET that are slightly different from *in vivo* pediatric values exerting a longer IT and a shorter ET.

### Tidal volume and respiratory minute-ventilation

TV values fluctuated from 33.16 ± 7.37 mL to 37.44 ± 7.43 mL and MV values ranged from 1.07 ± 0.21 L/min to 1.34 ± 0.31 L/min (Table 1). Results found in different papers showed that TV of infants suffering from BPD are often around 30mL^37,38^. Besides, in another study, they detected lower TV, around 15 mL, but this could be due to body weight of infants, that are lower than those of other studies^36^. Consequently, MV values obtained with the chimeric model are also in good accordance with data reported in the literature. Hence, the developed model showed TV and MV values very similar to *in vivo* data.

### Resistances and compliance

Lastly, R values are comprised between 253.5 ± 82.50cmH_2_O/L^−1^.s^−1^ and 290.1 ± 92.77cmH_2_O/ L^−1^.s^−1^, while C values covered the range from 1.78 ± 0.65 to 1.85 ± 0.99 mL/cmH_2_O (Table 1). R values are lower than 383 ± 36 cmH_2_O/ L^−1^.s^−1^ found in the study of Fok *et al*.^39^. C values are relatively lower in literature. C values are relatively lower in literature. Indeed, Greenspan *et al*.^40^ found C values around 1.51 ± 0.69 mL/cmH_2_O and Pfenninger *et al*.^41^ values were around 1.04 ± 0.42 mL/cmH_2_O. All things considered, R and C values seem to be in the same order of magnitude of *in vivo* pediatric data^39–44^. The chimeric model suffers from few limitations that could impact these results of compliance and resistance such as the lack of lung surfactant and the absence of blood perfusion. Indeed, surfactant is responsible for approximately 50% of compliance of the system^44^. Perfusion of lungs is also an important parameter related to resistances.

To sum up, all the 6 parameters assessed are found to be very similar to *in vivo* data reported in literature from infant or animal models frequently used as surrogates. However, due to the narrow dispersion of experimental data collected, significant differences can be observed for some of these 6 parameters measured when the breathing frequency varies (see Fig. 1). All things considered, the respiratory rate of 35 cycles/min appears to be the best compromise. To conclude, the *ex vivo* pediatric model developed in this study satisfactorily mimic breathing pattern and respiratory parameters of a BPD-suffering infant of 3 to 4 Kg of body weight.

### Assessment of ventilation by ^81m^krypton (^81m^Kr) scintigraphy

**Figure 2:**
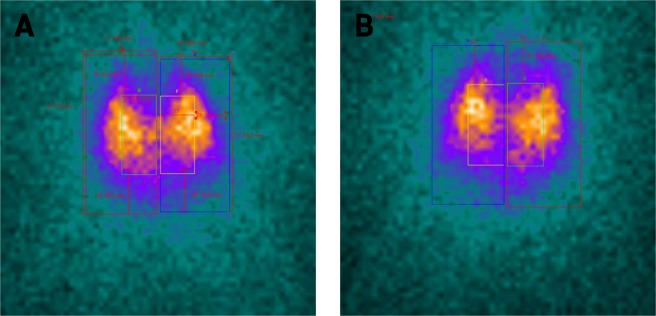
^81m^Krypton (^81m^Kr) scintigraphic images of lungs. (**A**) anterior view. (**B**) posterior view.

Scintigraphic measurements of ventilation using ^81m^Kr were made on 13 thoraxes. For these nuclear medicine experiments, the RR was set at 35 cycles/min as it appeared to be the best compromise. According to ^81m^Kr half-life, repartition of this gas within lungs is considered to be representative of regional ventilation^42^. The repartition of total count between right and left lung was studied and compared with pediatric *in vivo* studies^43,45,46^ (Fig. 2). Experiments showed an asymmetry in left/right regional ventilation with 56.94% ± 9.4% [51.23%; 62.65%] in right lung and 42.83% ± 9.36 [37.17%; 48.49%] with a significant difference between lungs (*p* = 0.019). Literature lack of quantitative data because ^81m^Kr ventilation scintigraphies are coupled with ^99m^Tc-albumin scintigraphy to assess ventilation/perfusion mismatch. Hence, comparison with available *in vivo* data was only qualitative. Ventilation repartition was similar to the one noticed in mild BPD^46^ with a higher proportion of signal in the right lung.

### Regional aerosol deposition

**Figure 3:**
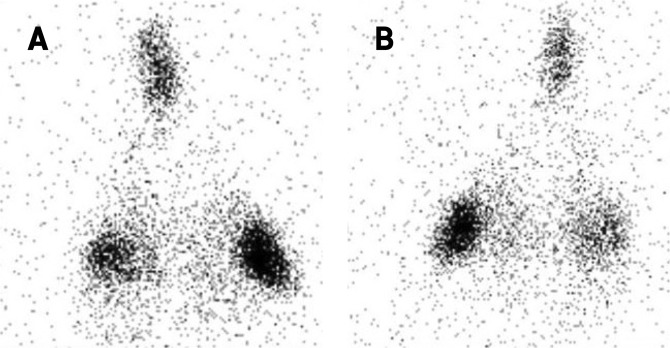
Diethylene triamine pentaacetic acid (^99m^Tc-DTPA) planar scintigraphic images of the *ex vivo* pediatric model developed in this study. (**A**) anterior view. (**B**) posterior view.

**Figure 4:**
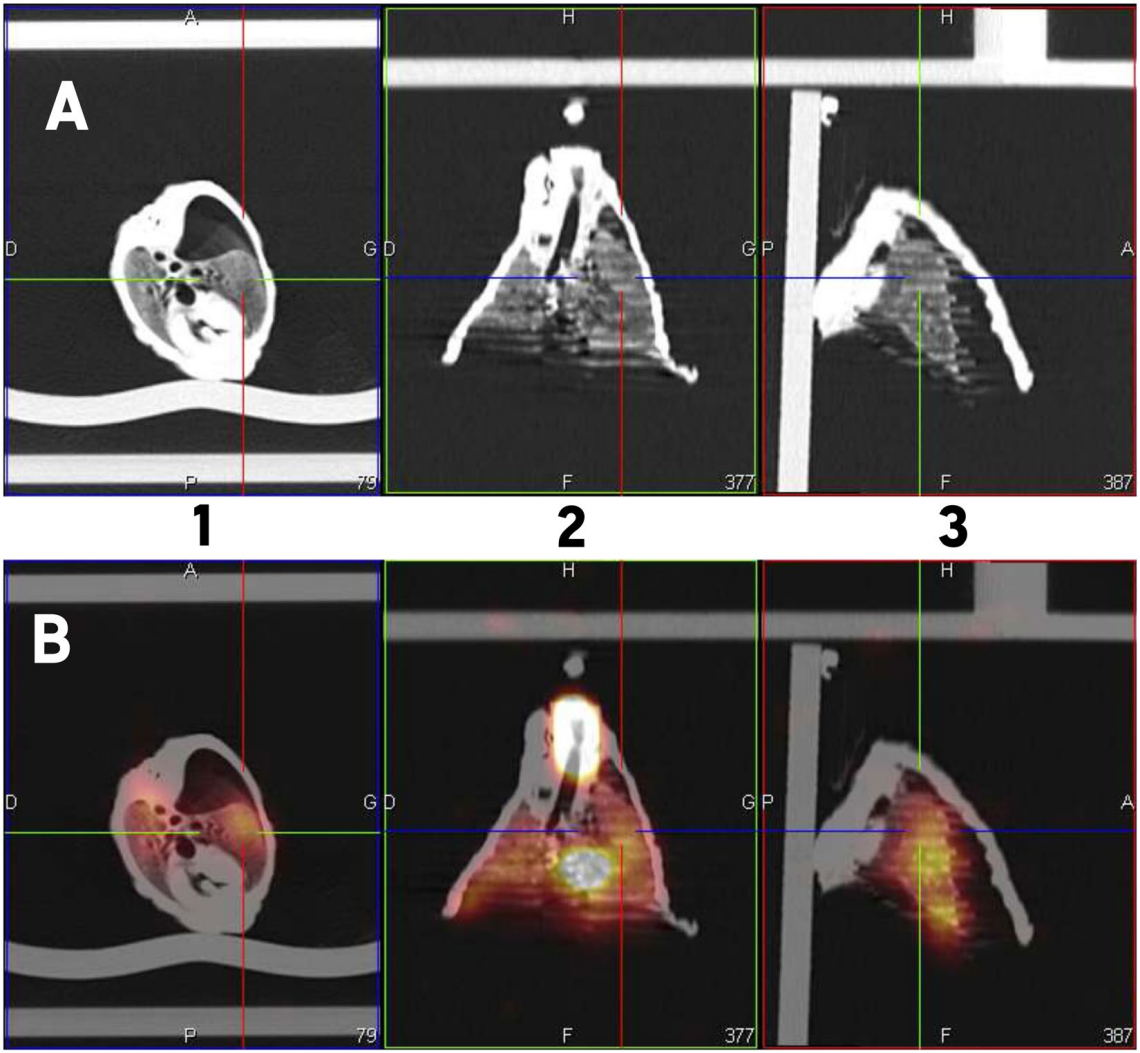
Diethylene triamine pentaacetic acid (^99m^Tc-DTPA) images after three dimension reconstruction of the *ex vivo* model developed in this study. (**A**) tomography images. (**B**) fusion of tomography and scintigraphic images. 1: transversal view. 2: coronal view. 3: sagittal view.

The regional aerosol deposition within the respiratory tract was assessed with ^99m^technetium complexed with diethylene triamine pentaacetic acid (^99m^Tc-DTPA) radioaerosol. The aerosol regional deposition was recorded by planar scintigraphy using gamma-camera imaging (*n* = 6, using 6 different respiratory models) and 3D scintigraphic images using SPECT-CT (*n* = 1). Three-dimensional images showed a repartition of nebulized dose in accordance with 2D results (Figs 3 and 4). Deposited fractions along nebulization system and within respiratory tracts were quantified and results are shown in Table 2. The different fractions are expressed as proportions of the initial activity in the nebulizer. Literature of such deposition studies *in vivo* is very scarce, even more considering pediatric *in vivo* studies. Most of studies did not use the same nebulizers as ours (Philips Sidestream^®^), which make results obtained in these studies difficult to compare to our own. Moreover, most of studies focused on intubated infants or surrogate animal models that represents an important bias. Table 3 reports data available in literature. Only studies using a jet nebulizer were selected to allow relevant comparison with these data produced using the chimeric model. Results demonstrated that lung deposited fraction in the *ex vivo* chimeric model is in good accordance with *in vivo* data for 3-months old infants. Therefore, the developed model appears to be an interesting tool to successfully assess the regional deposition of aerosol for pediatric model of BPD-suffering infants.

**Table 2:** Deposited fractions along nebulization system as a proportion of initial activity in the nebulizer (mean ± SD, n = 6).

Nebulized activity	Interface	Replica	Expiratory filter	Lungs	Pump filter
50.33 ± 6.75%	7.82% ± 6.91%	15.34% ± 10.20%	23.57% ± 13.87%	2.60% ± 2.24%	1.00% ± 1.38%

Interface: naso-buccal mask. Replica: 3D-printed infant nasal replica with connecting tube. Expiratory filter: collection of exhaled aerosols. Pump filter: security filter to avoid contamination of the depression generator.

**Table 3:** Comparison of published studies using jet nebulizers in pediatric modelss. NA: not available.

Study	Model	Age	Weight	Nebulizer	Lung deposited fraction
Montigaud *et al*.	*Ex vivo* chimeric model	Around 3 months	Between 3–4Kg	Philips Sidestream	2.60% ± 2.24%
Watterberg *et al*.^51^	Intubated infants/toddlers	9–36 months	NA	Travenol #2C7161	≤1%
Fok *et al*.^52^	Intubated infants	Around 2.5 months	Around 1.7Kg	MedicAid Sidestream	0.4 to 2.62%
	Non-intubated infants	Around 3 months	Around 2.4Kg		0.87 to 3.43%
Fok *et al*.^53^	Ventilated rabbits	NA	Around 3Kg	Hudson RCI Flo Thru®	3.34%
Dubus *et al*.^27,28^	Ventilated macaques	44 months	2.5 to 2.8Kg	MistyNeb	0.4 to 1.4%
	Ventilated piglets	2 days	1.7Kg ± 0.3Kg		0.50 to 7.70%
Cameron *et al*.^54^	Ventilated rabbits	NA	1.5Kg	Mallinckrodt Ultravent®	2.8%

However, this model raises few limitations, such as the supine position during nebulization process that decreases penetration of airborne particles and obviously the lack a physiopathological features of the disease. Thus, regional aerosol deposition could be slightly modified.

## Conclusion

BPD induced mortality, as one of the most common diseases of preterm infants, decreased since neonatal care unit became more and more qualified. However, new techniques induced a modification of the physiopathology and morbidity of this disease partly due to side effects of mechanical ventilation and oxygen supplementation. In response, some drugs are used to wean prematurely born infants from ventilation or oxygen supplementation but they have adverse effects. Inhaled therapies could be an interesting route of administration for such treatments, aiming the specifically defective organ. However, uneven and/or uncontrolled deposition of drugs along respiratory tract is one the remaining challenge to improve inhaled therapies.

In this study, an *ex vivo* respiratory model of BPD was developed to assess aerosol regional deposition. This chimeric model consists in a 3D-printed infant upper airways replica connected to a leporine thorax placed in a sealed enclosure producing pleural depression. This method mimic passive ventilation by inducing negative pressure within the enclosure. Breathing pattern and parameters are easily and reproducibly controllable. Three RR were tested (*i*.*e*. 30, 35 and 40 cycles/min) that led to experimentally measure 6 parameters. Results obtained clearly demonstrated that the breathing pattern obtained for the chimeric model was very similar compared to *in vivo* data of 3-months old BPD-suffering infants available in the literature. Then, using ^81m^Kr planar scintigraphies, homogeneity of ventilation of the developed model was proved to be comparable to ventilation behavior observed in infants. Lastly, a regional aerosol deposition with ^99m^Tc-DTPA planar and 3D SPECT/CT experiments was performed. The results obtained were analogous to those found for *in vivo* studies from the literature. However, the developed model suffers from limitations, among others, the supine position and the lack of lung surfactant and blood perfusion. To counterbalance these limitations, this *ex vivo* chimeric model presents many advantages. It perfectly fits the 3R guidelines (Refine, Reduce and Replace) as a good and relevant surrogate model to animal experiments. Secondly, this model is relatively costs saving. Indeed, it does not need any animal husbandry (nor animals). Lastly, this model is easy to use and could be tuned as wanted. Hence, this study demonstrated that this new *ex vivo* model could reliably simulate BPD with less ethical restrictions and allowing an alternative to animal experiments.

## Materials and Methods

### Materials

Upper airways and larynx are composed of a stereolithographied infant nasal replica obtained by reconstruction from CT scan images of a SAINT model^47^ and by three-dimensional printing technology. It integrated laryngeal structures to mimic vocal folds resistances. This 3D-printed replica is well adapted and was previously used for nasal and pulmonary deposition studies^26,47–49^. Rabbit thoraxes are obtained from leporine slaughterhouses (Rouget Volailles, La Talaudière France), satisfying French sanitary controls, and were used within 24 hours or frozen at −20 °C depending on availability. Visual control of wound was performed and assessment of recruitment of each lung was carried out. Thoraxes were placed in a sealed enclosure and ventilated using a depression generator SuperDimension^®^ (Covidien, Dusseldorf, Germany) mimicking pleural depression similarly to *in vivo* passive ventilation. A picture of the setup is available online as Supplementary Figs S1 and S2.

Forty leporine thoraxes were used to analyze respiratory parameters. Twelves of them (30%) were used within 48 h, others were frozen and thawed at least 12 hours before experiments due to supplier unavailability. Among 43 thoraxes, 5 (5%) were excluded due to major lung injuries that impaired ventilation. Thoraxes were not used for more than 8 hours. A video of ventilating model is available as Supplementary Video SV1. Before slaughter, rabbits were around 3Kg of bodyweight.

The 3R guidelines (Refine, Reduce and Replace) are 50 years old framework focused on ethical considerations of animal use in research. It defines ways to reduce animal suffering and increase reliability of produced data. Moreover, it recommends using substitutive methods to animal experiments. As our *ex vivo* model uses wastes of food industry instead of live animals, it fits in these guidelines and represents a good alternative to animal experiments.

### Passive ventilation assessment

Instrumentation of the enclosure was realized with a Biopac^®^ system (Biopac, Goleta, USA) composed of a pneumotachograph (TSD 117) and a differential pressure transducer (TSD160D), which are connected to amplifier (DA100C) plugged on unit acquisition (M160). This system allowed a real-time follow-up of depression in the enclosure and airflow at the replica. AcqKnowledge^®^ 5.0 software was used to determine respiratory parameters for each cycle: peak inspiratory flow (PIF), peak expiratory flow (PEF), tidal volume (TV), minute-ventilation (MV), respiratory rate (RR), inspiratory time (IT), expiratory time (ET), total time (TT), resistances (R) and compliance (C).

Data were recorded for 3 minutes with fixed IT of 0.4 s and tuned ET to reach 30, 35 and 40 respiratory rates (1.6 s, 1.3 s and 1.1 s respectively). Average, standard deviation and 95% confidence interval were calculated for at least 50 cycles to obtain a representative values for each thorax. Breathing patterns and parameters were chosen to represent infants with BPD at rest.

### Ventilation and regional aerosol deposition

Regional ventilation was assessed using ^81m^krypton (^81m^Kr) scintigraphy^50^ while regional aerosol deposition was observed with ^99m^technetium complexed with diethylene triamine pentaacetic acid (^99m^Tc-DTPA) scintigraphy. Aerosol was generated from a 100MBq in 3 mL solution of ^99m^Tc-DTPA using a Sidestream^®^ jet nebulizer (Philips Healthcare, Suresnes, France) connected to the infant nasal replica with a naso-buccal mask (Ambu^®^, Bordeaux, France).

For ventilation scintigraphies, an acquisition was made for each RR with a naso-buccal mask. Two different regions of interest (ROI) were identified on ventilation scintigraphies to define left and right lung. Additional details about the method are provided in the online data supplement. Scintigraphic measurements of ventilation using ^81m^Kr were made on 14 thoraxes but 1 (7.1%) had to be censored due to obstruction of a primary bronchi leading to biased repartition of the gaz.

For aerosol regional deposition study, 6 thoraxes were used for 2D scintigraphies. These planar scintigraphic images (matrix 256*256) were recorded with a variable angle dual detector Single Photon Emission Computed TomographY/Computed Tomography(SPECT/ CT, SYMBIA T2; Siemens, Knoxville, TN) equipped with a low-energy, high-resolution collimator (FWHM 8.3 mm at 10 cm); tested weekly for uniformity (UFOV 533 mm × 387 mm, CFOV 400 mm × 290 mm). Before conducting the inhalation experiments, the initial radioactive doses filled in the nebulizer were quantified (scintigraphic images, 60-sec anterior/posterior, were acquired corresponding to the full and empty syringe). Once the inhalation experiments were performed, 180-sec anterior/posterior images of the experimental setup were acquired for each element: empty nebulizer, expiratory filter, infant nasal replica and lungs. An interest ROI was delimited on the images with a correction of the background radiation using the mean of three external ROIs. Results were expressed in terms of the activity loaded into the nebulizer.

After acquiring 2D images with the same gamma camera (SYMBIA T2; Siemens, Knoxville, TN), SPECT and CT acquisitions were performed immediately for attenuation correction and anatomical mapping. A 3D SPECT acquisition of the lungs was performed with 64 (2 × 32) projection images, each 30 s. Finally, a CT was performed with the following parameters: 130 kV, 90mAs, 1.25 mm slice thickness, 0.9 mm increment, 1.6 mm pitch, and rotation time of 1.5 s. A multimodality computer platform (Symbia net; Siemens) was used for image reviews and manipulations. Both the transmission and emission scans were reconstructed using 3D OSEM by default (8 subsets, 5 iterations), with pre-reconstruction smoothing using a 3D Butterworth filter (cutoff: 0.45 cycles/cm; order 5), a 128 × 128 image matrix, a 1.23 zoom, and a pixel size of 3.9 mm. SPECT images were reconstructed using scatter correction (scatter energy window) and CT attenuation correction. CT and SPECT images were matched and fused into trans-axial images. The tracheobronchial area included the trachea and the left and right main bronchi. There, 1 thorax was used to collect data.

### Statistical analyses

Results are expressed as mean ± standard deviation (95% confidence interval). For respiratory parameters, Gaussian distribution was assessed with a Shapiro-Wilk normality test. If Gaussian distribution was validated, a repeated-measure one-way ANOVA with Tukey’s multiple comparison *post hoc* test was used. If distribution was not Gaussian, Friedmann’s test with Dunn’s multiple comparison *post hoc* test was performed. For ventilation studies, a paired t-test was used to compare left and right lung fraction of total counted radioactivity. All tests were two-sided and *p* < 0.05 was considered statistically significant. Statistical analyses were performed using GraphPad Prism® 7 (GraphPad Software, La Jolla, CA, USA).

## Supplementary information

Supplementary Figures

Supplementary Video SV1
