# Supplementary material for: Development of an ex vivo respiratory pediatric model of bronchopulmonary dysplasia for aerosol deposition studies
Source: Sci Rep. 2019 Apr 5;9:5720. doi: 10.1038/s41598-019-42103-2 (PMC6450907; doi:10.1038/s41598-019-42103-2)
Supplement: Supplementary file 1 — Supplementary Figures [file 41598_2019_42103_MOESM1_ESM.pdf]

Development of an *ex vivo* respiratory pediatric model of bronchopulmonary dysplasia for aerosol deposition studies

Yoann Montigaud[1], Sophie Périnel[2,3], Jean-Christophe Dubus[4], Lara Leclerc[1], Marie Suau[1], Clémence Goy[2,3], Anthony Clotagatide[2,3], Nathalie Prévôt[2,3], Jérémie Pourchez[1]

[1]Mines Saint-Etienne, Univ Lyon, Univ Jean Monnet, INSERM, U 1059 Sainbiose, Centre CIS, F - 42023 Saint-Etienne France.
[2]INSERM U 1059 Sainbiose, Université Jean Monnet, Saint-Etienne, F-42023, France.
[3]CHU Saint-Etienne, Saint-Etienne, F-42055, France.
[4]Médecine infantile, pneumo-allergologie, CRCM & CNRS, URMITE 6236, Assistance publique-Hôpitaux de Marseille, 13385 Marseille cedex 5, France.

Correspondence and requests for materials should be addressed to J.P. (email: pourchez@emse.fr)

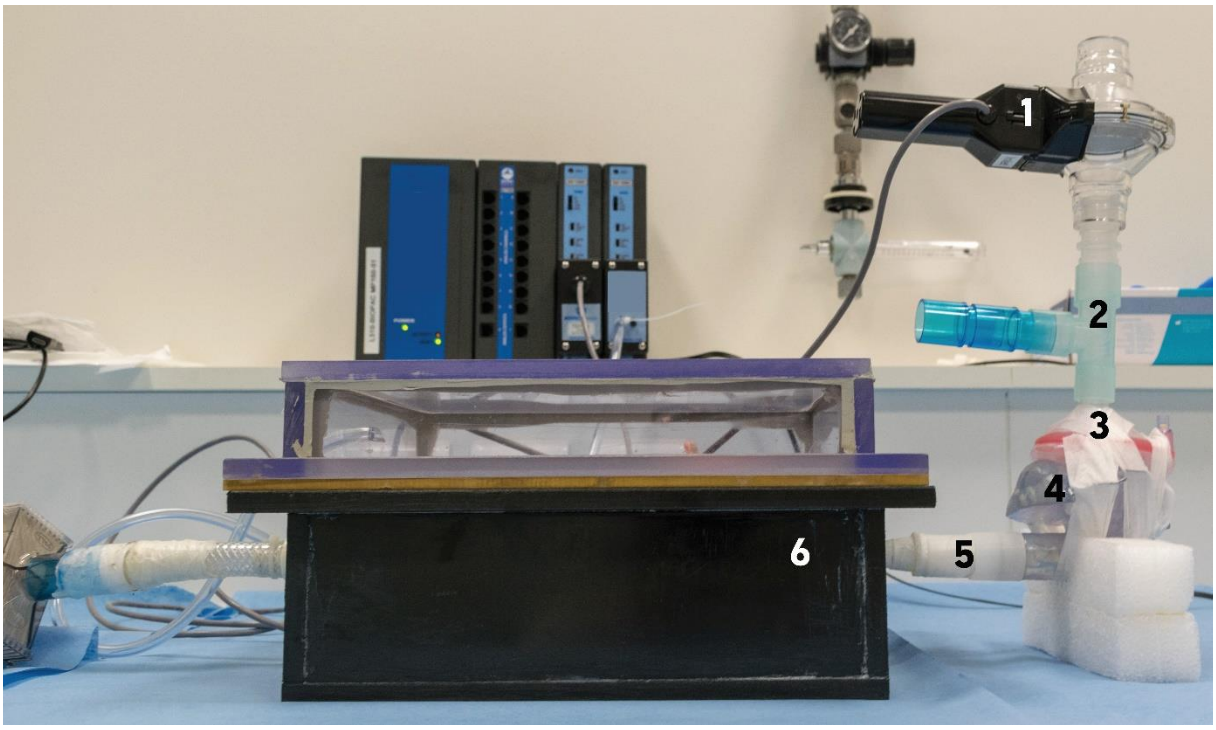

Figure S1 - Image of the setup used to acquire physiological measurements. 1: pneumotachograph. 2: connecting tube. 3: mask. 4: SAINT model replica. 5: connecting tube. 6: sealed enclosure.

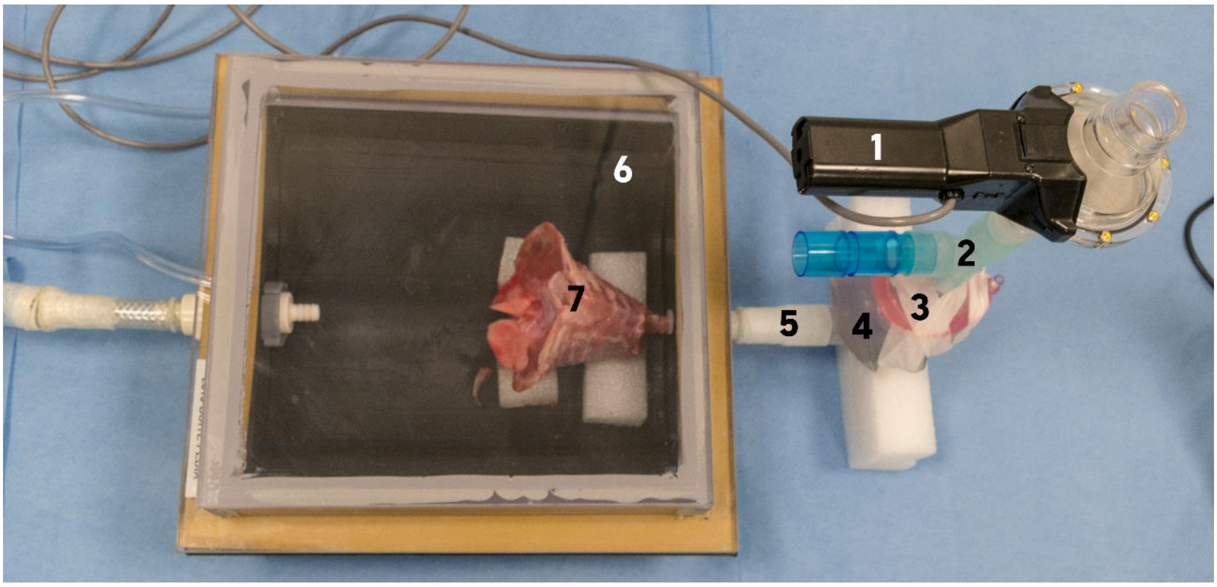

Figure S2 - Image of the setup used to acquire physiological measurements. 1: pneumotachograph. 2: connecting tube. 3: mask. 4: SAINT model replica. 5: connecting tube. 6: sealed enclosure. 7: leporine thorax.
